# *PIK3CA* activating mutations are associated with more disseminated disease at presentation and earlier recurrence in glioblastoma

**DOI:** 10.1186/s40478-019-0720-8

**Published:** 2019-04-29

**Authors:** Shota Tanaka, Tracy T. Batchelor, A. John Iafrate, Dora Dias-Santagata, Darrell R. Borger, Leif W. Ellisen, Daniel Yang, David N. Louis, Daniel P. Cahill, Andrew S. Chi

**Affiliations:** 1 Stephen E. and Catherine Pappas Center for Neuro-Oncology, Department of Neurology, Boston, USA; 2 Translational Research Laboratory, Cancer Center, Boston, USA; 3 Department of Pathology, Boston, USA; 4 Department of Neurosurgery, Boston, USA; 5 Massachusetts General Hospital Cancer Center, Harvard Medical School, 55 Fruit Street, Yawkey 9E, Boston, MA 02114 USA; 6 The University of Tokyo Hospital, Tokyo, Japan; 7 Present Address: Brigham and Women’s Hospital, Harvard Medical School, Boston, MA USA; 8 Present Address: Dana-Farber Cancer Institute, Harvard Medical School, Boston, MA USA; 9 Perlmutter Cancer Center, New York University Langone Health and School of Medicine, New York, USA; 10 Present Address: Neon Therapeutics, 40 Erie Street, Suite 110, Cambridge, MA USA

**Keywords:** Dissemination, Glioblastoma, Gliomatosis, Multicentric, *PIK3CA*

## Abstract

Phosphatidylinositol 3-kinase signaling promotes cell growth and survival and is frequently activated in infiltrative gliomas. Activating mutations in *PIK3CA* gene are observed in 6–15% of glioblastomas, although their clinical significance is largely undescribed. The objective of this study was to examine whether *PIK3CA* mutations are associated with a specific clinical phenotype in glioblastoma. We retrospectively reviewed 157 consecutive newly diagnosed glioblastoma patients from December 2009 to June 2012 who underwent molecular profiling consisting of targeted hotspot genotyping, fluorescence in situ hybridization for gene amplification, and methylation-specific PCR for O^6^-methylguanine-DNA methyltransferase promoter methylation. Molecular alterations were correlated with clinical features, imaging and outcome. The Cancer Genome Atlas data was analyzed as a validation set. There were 91 males; median age was 58 years (range, 23–85). With a median follow-up of 20.9 months, median progression-free survival (PFS) and estimated overall survival (OS) were 11.9 and 24.0 months, respectively. Thirteen patients (8.3%) harbored *PIK3CA* mutation, which was associated with younger age (mean 49.4 vs. 58.1 years, *p* = 0.02). *PIK3CA* mutation correlated with shorter PFS (median 6.9 vs. 12.4 months, *p* = 0.01) and OS (median 21.2 vs. 24.2 months, *p* = 0.049) in multivariate analysis. A significant association between *PIK3CA* mutation and more disseminated disease at diagnosis, as defined by gliomatosis, multicentric lesions, or distant leptomeningeal lesions, was observed (46.2% vs. 11.1%, *p* = 0.004). In conclusion, despite the association with younger age, *PIK3CA* activating mutations are associated with earlier recurrence and shorter survival in adult glioblastoma. The aggressive course of these tumors may be related to their propensity for disseminated presentation.

**Electronic supplementary material:**

The online version of this article (10.1186/s40478-019-0720-8) contains supplementary material, which is available to authorized users.

## Introduction

Recent years have witnessed a remarkable transformation in our understanding of glioma development and classification. It is now recognized that gliomas consist of clinically distinct, molecularly-defined disease entities, with different entities often distinguished by somatic mutations in single genes [1–3]. These molecular subgroups are not only distinguishable by their biology and associations with outcome, but many have highly distinct clinical characteristics such as age at diagnosis, radiographic appearance, or location within the central nervous system [4–6]. However, there remains variability within glioma molecular subgroups, and identification of novel genotype-phenotype correlations may allow for further refinement of molecular classification and improve the development of novel therapies [7, 8].

Class I_A_ phosphatidylinositol 3-kinase (PI3K) is activated in several cancer types by somatic activating hotspot mutations in the phosphatidylinositol-4,5-bisphosphate 3-kinase catalytic subunit alpha (*PIK3CA*) gene, which encodes the catalytic subunit p110α. In some cases, somatic truncations/in-frame deletions of the phosphatidylinositol-4,5-bisphosphate 3-kinase regulatory subunit 1 (*PIK3R1*) gene, which encodes the regulatory subunit p85α, are also observed [9, 10]. In vitro and in vivo, *PIK3CA* and *PIK3R1* mutations constitutively increase PI3K pathway activity, and are oncogenic in several cancer models [11–14].

Recently, recurrent somatic mutations in *PIK3CA* and *PIK3R1* were identified in 6–15 and 10% of glioblastomas, respectively [15, 16], which were accompanied by activated PI3K signaling [15]. However, the clinical impact of these mutations is largely undescribed in glioblastoma. Therefore, we sought to determine whether somatic mutations in *PIK3CA* are associated with a distinct phenotype in patients with newly diagnosed glioblastoma.

## Patients and methods

### Patients and tumor specimens

We retrospectively analyzed a consecutive cohort of adult patients with newly diagnosed glioblastoma that had been molecularly profiled in our center from December 2009 to June 2012 (*n* = 184). We included only patients with newly diagnosed glioblastoma with a Karnofsky performance status (KPS) score of at least 60 and who were treated with standard chemoradiation (*n* = 157) [17]. Patients with a known history of lower grade glioma were excluded.

Medical charts and images were retrospectively reviewed to gather data on patient and tumor characteristics as well as the type of surgery. The extent of resection was assessed based on T1-weighted images with gadolinium enhancement. We defined widespread disseminated disease as having either a diffuse gliomatosis-like growth pattern (tumor in three or more lobes, including the brain stem), multicentric lesions (lesions in different lobes or > 2 cm apart without intervening T2/fluid-attenuated inversion recovery changes), or distant leptomeningeal lesions.

### Genotyping

Molecular profiling included SNaPshot genotyping, which interrogates 68 established hotspot loci from 15 oncogenes and tumor suppressors (*AKT1, APC, BRAF, CTNNB1,* epidermal growth factor receptor (*EGFR*)*, HER2,* isocitrate dehydrogenase 1 (*IDH1*)*, KIT, KRAS, MEK1, NOTCH1, NRAS, PIK3CA, PTEN, TP53*), fluorescence in situ hybridization (FISH) for *EGFR*, mesenchymal-epithelial transition (*MET*), and platelet-derived growth factor receptor alpha (*PDGFRA*) gene amplifications, and methylation-specific PCR for O^6^-methylguanine-DNA methyltransferase (*MGMT*) promoter methylation [18]. *PIK3R1*, *IDH2*, and *TERT* promoter were not included in this genotyping platform.

### Survival analysis

In this retrospective analysis, progressive disease was defined either by tissue diagnosis or when two of the following criteria were met: a) radiographic progression by central review that occurred after more than 3 months from the end of radiation, b) neurological decline related to the tumor (clinical progression) documented by the treating physician, and c) initiation of new anti-tumor therapy. Overall survival (OS) was calculated from the day of initial surgery. Patients were censored when they were lost to follow-up or died from causes unrelated to the disease.

### Independent dataset validation

Glioblastoma mutation and copy number data from The Cancer Genome Atlas (TCGA) datasets were downloaded from www.cbioportal.org [19–21]. The mutation and progression-free survival (PFS) data of 291 sequenced glioblastomas in the TCGA project [19] were accessed on September 22, 2017 and used for survival analysis. Kaplan-Meier curves for PFS were calculated with stratification by *PIK3CA* mutation status.

### Statistical analysis

Two-tailed Student’s t-test and Fisher’s exact test were used to compare continuous and categorical variables between two groups, respectively. The log-rank test was used in univariate analysis of factors associated with survival. The Cox hazards model was used in multivariate analysis. Age and KPS score were assessed as continuous variables. JMP 11 (SAS Institute, Cary, NC, USA) was used for statistical analysis. A probability (*p*) value of < 0.05 was considered significant.

## Results

### Patient characteristics, survival, and established prognostic factors in the cohort

A total of 157 consecutive adult patients with molecularly profiled glioblastoma who had a KPS score of at least 60 and were treated with standard chemoradiation were included in the analysis. The median age was 58 years (range, 23–85), there were 91 males (58.0%) and the median KPS score was 90 (Table 1). Most patients had undergone resection (136 patients, 86.6%) rather than only biopsy, and 69 (44.0%) patients underwent gross total resection (GTR) of enhancing disease. With a median follow-up of 20.9 months for the entire cohort, the median PFS and OS of the entire cohort were 11.9 and 24.0 months, respectively. As expected, established clinical prognostic factors such as age, KPS, and GTR were associated with longer PFS and/or OS in univariate analyses (age: PFS *p* = 0.0004, OS *p* = 0.0004; KPS: PFS *p* = 0.10, OS *p* = 0.047; GTR: PFS *p* = 0.02, OS *p* = 0.11) (Table 2). Of note, deep tumor location (i.e. basal ganglia or corpus callosum) compared to hemispheric location was not associated with either PFS (*p* = 0.50) or OS (*p* = 0.65), and therefore was not included in further survival analyses. Similarly, the known favorable molecular prognostic factors of *IDH1* mutation and *MGMT* promoter methylation, observed in 7.6% (12/157 tumors) and 50.0% (60/120 tumors), respectively, were also associated with longer PFS and OS (*IDH1*: PFS *p* < 0.0001, OS *p* = < 0.0001; *MGMT*: PFS *p* < 0.0001, OS *p* < 0.0001) (Table 2, Additional file 1: Figure S1).

**Table 1:** Patient and tumor characteristics

	Total	*PIK3CA* mutant	*PIK3CA* wildtype	*P* value
Number of patients	157	13	144	
(male patient)	(91 male)	(9 male)	(82 male)	0.56
Age	median 58 (23–85)	mean 49.4 ± 15.9	mean 58.1 ± 12.1	0.02^*^
KPS score	median 90 (60–100)	mean 92.3 ± 7.3	mean 87.7 ± 10.4	0.048^*^
Gross total resection	69 (44.0%)	5 (38.5%)	64 (44.4%)	0.78
*IDH1* mutation	12/157 (7.6%)	0/13 (0.0%)	12/144 (8.3%)	0.60
*MGMT* promoter methylation	60/120 (50.0%)	8/11 (72.7%)	52/109 (47.7%)	0.20
*EGFR* amplification	63/143 (44.1%)	2/12 (16.7%)	61/131 (46.6%)	0.07

* Statistical significance: *P* ≤ 0.05

Abbreviations: KPS, Karnofsky performance status; *IDH1*, isocitrate dehydrogenase 1; *MGMT* O^6^-methylguanine-DNA methyltransferase

**Table 2:** *PIK3CA* mutation was significantly associated with shorter survival after adjusting for age, KPS, gross total resection, *IDH1* mutation, and *MGMT* promoter methylation

	Univariate analysis	Multivariate analysis	
	*P* value	*P* value	HR
[PFS]			
High age	0.0004^*^	0.005^*^	1.03 (1.01–1.05)^#^
High KPS score	0.10	0.28	0.99 (0.97–1.01)^#^
Gross total resection	0.02^*^	0.17	0.75 (0.49–1.13)
*PIK3CA* mutation	0.03^*^	0.01^*^	2.89 (1.30–5.90)
*IDH1* mutation	< 0.0001^*^	0.002^*^	0.10 (0.006–0.50)
*MGMT* promoter methylation	< 0.0001^*^	< 0.0001^*^	0.33 (0.21–0.52)
[OS]			
High age	0.0004^*^	0.003^*^	1.03 (1.01–1.05)^#^
High KPS score	0.047^*^	0.32	0.99 (0.96–1.01)^#^
Gross total resection	0.11	0.53	0.87 (0.55–1.35)
*PIK3CA* mutation	0.19	0.049^*^	2.32 (1.00–4.88)
*IDH1* mutation	< 0.0001^*^	0.02^*^	0.15 (0.008–0.74)
*MGMT* promoter methylation	< 0.0001^*^	< 0.0001^*^	0.27 (0.17–0.44)

* Statistical significance: *P* ≤ 0.05

^#^ HR per unit

Abbreviations: PFS, progression-free survival; OS, overall survival; KPS, Karnofsky performance status; *IDH1*, isocitrate dehydrogenase 1; *MGMT*, O^6^-methylguanine-DNA methyltransferase; HR, hazard ratio

### Characteristics of PIK3CA mutant glioblastoma

**Fig. 1:**
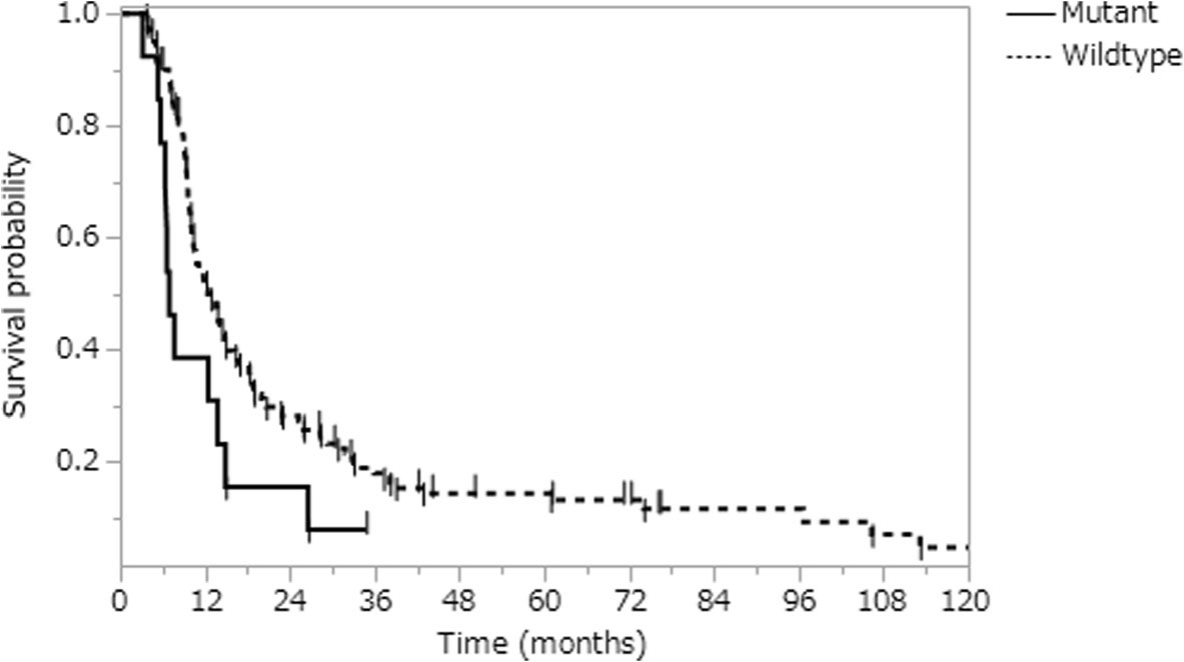
Kaplan-Meier curves of PFS stratified by *PIK3CA* mutation. *PIK3CA* mutant tumors (solid) and wildtype tumors (dashed) depicted

We identified 13 patients (8.3%) with *PIK3CA* activating mutations by DNA sequence analysis (4 at position R88, 4 at hotspots 542–546, and 5 at position H1047) (Table 3). *PIK3CA* mutation was significantly associated with a younger age (*p* = 0.02) and better KPS (*p* = 0.048) (Table 1). Strikingly, it was also associated with decreased PFS (median 6.9 months for mutant vs. 12.4 months for wildtype, *p* = 0.03) (Fig. 1), an association that remained significant after adjusting for other known prognostic factors in a multivariate model, including age, KPS, GTR, *IDH1* mutation, and *MGMT* promoter methylation (HR 2.89, *p* = 0.01). There was no difference in OS in univariate analysis (median 21.2 months vs. 24.2 months, *p* = 0.19) (Additional file 2: Figure S2); however, OS was significantly worse in *PIK3CA* mutant patients in multivariate analysis after adjusting for the above known prognostic factors (HR 2.32, *p* = 0.049) (Table 2). Other factors that were independently associated with survival in multivariate analysis were high age (PFS: HR 1.03, *p* = 0.005; OS: HR 1.03, *p* = 0.003), *IDH1* mutation (PFS: HR 0.10, *p* = 0.002; OS: HR 0.15, *p* = 0.02), and *MGMT* promoter methylation (PFS: HR 0.33, *p* < 0.0001; HR 0.27, *p* < 0.0001) (Table 2).

**Table 3:** Case description of *PIK3CA*-mutant glioblastomas

Case	Age	Sex	Tumor at presentation	Type of surgery	KPS	*PIK3CA* mutation	*MGMT*	*IDH1*	*EGFR*	Other alterations	PFS (mo.)	OS (mo.)
1	67	M	multicentric	PR	90	H1047R	u	wt	n.t.	*TP53* R248W	6.3	9.7
2	68	M	gliomatosis	biopsy	90	R88Q	n.t.	wt	N		3.2	5.1
3	31	M	leptomeningeal metastasis	biopsy	90	H1047R	n.t.	wt	N		6.6	10.9
4	42	F	single lesion	PR	100	R88Q	m	wt	N	*TP53* R273C	12.4	54.9
5	72	F	single lesion	GTR	90	R88Q	m	wt	N		5.3	10.6
6	35	F	multicentric	GTR	90	Q546R	u	wt	amp		26.6	32.4
7	25	M	multicentric	biopsy	80	R88Q	m	wt	N		6.9	19.1
8	65	F	single lesion	GTR	100	Q546K	m	wt	N		7.6	36.9
9	43	M	single lesion	PR	90	E545K	u	wt	N		5.7	11
10	32	M	single lesion	PR	100	H1047R	m	wt	N		13.8	17.7*
11	54	M	single lesion	GTR	100	E542K	m	wt	N		34.8*	34.8*
12	57	M	gliomatosis	biopsy	80	H1047Y	m	wt	N		6.6	21.2
13	51	M	single lesion	GTR	100	H1047R	m	wt	amp		14.9	31.1

Abbreviations: M, male; F, female; PR, partial resection; GTR, gross total resection; KPS, Karnofsky performance status; n.t., not tested; *IDH1*, isocitrate dehydrogenase 1; *MGMT*, O^6^-methylguanine-DNA methyltransferase promoter methylation; m, methylated; u, unmethylated; wt, wild-type; amp, amplified; N, non-amplified; PFS, progression-free survival; OS, overall survival; mo., months; *censored at last follow-up

Given the known prognostic impact of *IDH1* mutation on survival, we investigated the association of *PIK3CA* mutation with PFS and OS after excluding 12 *IDH1* mutant glioblastomas. Within the remaining 145 *IDH1* wildtype glioblastoma patients, the difference in PFS between *PIK3CA* mutant and *PIK3CA* wildtype tumors was statistically significant by Wilcoxon test (*p* = 0.02) and was a trend to significance by log-rank test (*p* = 0.11) (Additional file 3: Figure S3). In a multivariate analysis adjusting for other known prognostic factors such as age, KPS score, GTR, and *MGMT* promoter methylation, *PIK3CA* mutation was associated with decreased PFS (HR 2.85, *p* = 0.01) (Additional file 6: Table S1). With regards to OS, we observed no difference by *PIK3CA* mutation in univariate analysis (median 21.2 months vs. 22.2 months, *p* = 0.42); however in multivariate analysis there was a trend for *PIK3CA* mutant tumors to have worse OS (HR 2.25, *p* = 0.057) (Additional file 6: Table S1, Additional file 4: Figrue S4).

### PIK3CA mutation in the TCGA dataset

To confirm the impact of *PIK3CA* mutation on patient survival observed in our discovery cohort, we analyzed the publicly available validation dataset derived from the TCGA project [20, 21]. *PIK3CA* mutations were observed in 26 of 291 (8.9%) glioblastomas in this cohort. PFS of the patients with *PIK3CA* mutant glioblastoma (6.1 months) was significantly shorter than that of patients with the wildtype counterpart (9 months) (*p* = 0.008) (Additional file 5: Figure S5), while OS was not significantly different (median 13.1 months vs. 13.3 months, *p* = 0.40). Of note, *PIK3R1* mutations were noted in 25 of 256 (10.0%) glioblastomas; however, these mutations were not significantly associated with shorter PFS (median 6.2 months vs. 8.5 months, *p* = 0.14) or OS (median 13.7 months vs. 13.3 months, *p* = 0.73).

### Imaging characteristics for PIK3CA mutant glioblastomas

**Fig. 2:**
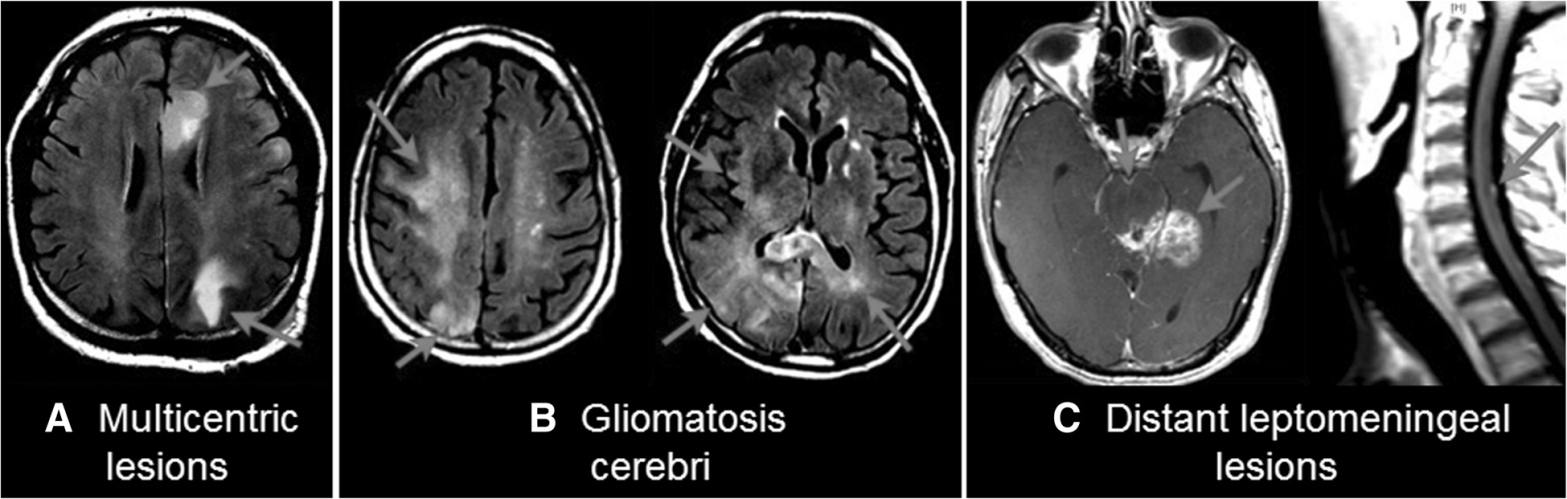
Representative cases of *PIK3CA* mutant glioblastomas demonstrating widespread disease at presentation. **a** The tumors were multicentric, affecting the left frontal and left parietal lobes without any intervening T2/fluid attenuated inversion recovery abnormality. **b** The tumor diffusely involved multiple lobes including the right frontal, parietal, and occipital lobes as well as the left parietal lobe. **c** The main tumor was located in the left posterior mesial temporal lobe with leptomeningeal involvement of the adjacent brain such as the midbrain and pons. Distant lesions were also observed on the ventral surface of the pons and the dorsal surface of the cervical spinal cord

To identify the putative causes of the apparent aggressive behavior of *PIK3CA* mutant tumors, we reviewed their clinical profiles. *PIK3CA* mutant glioblastoma often presented with widespread tumor dissemination relative to *PIK3CA* wildtype tumors. Representative contrast-enhanced MRI images of three *PIK3CA* mutant cases are shown in Fig. 2, with arrows indicating the tumor areas. We compared *PIK3CA* mutant and wildtype tumors for dissemination at diagnosis, defined as having either a diffuse gliomatosis-like growth pattern, multicentric lesions, or distant leptomeningeal lesions. We found a significant association between *PIK3CA* mutation and more disseminated disease at diagnosis, as 6 of 13 (46.2%) *PIK3CA* mutant glioblastomas presented with dissemination compared to 16 of 144 (11.1%) *PIK3CA* wildtype tumors (*p* = 0.004).

We also examined the effects of other molecular alterations associated with glioblastoma migration. High-level *EGFR* amplification was previously detected in the invasive subpopulation within heterogeneously amplified glioblastomas [22, 23]. In our dataset, 63 of 144 (43.8%) tested tumors showed high-level *EGFR* amplification. We did not observe a significant association between *EGFR*-amplification and dissemination (20.6% of *EGFR*-amplified tumors vs. 16.0% of non-amplified tumors, *p* = 0.52). Half of the tumors tested had *MGMT* promoter methylation (59/118, 50%), and there was no association with dissemination (18.6% of methylated tumors vs. 13.6% of unmethylated tumors, *p* = 0.62). After standard chemoradiation, tumors with *MGMT* promoter methylation were reported to have higher rates of distant recurrence [24]. No molecular alterations were associated with dissemination in the newly diagnosed setting when tested among the *PIK3CA* wildtype tumors alone (data not shown).

## Discussion

Here, we found that *PIK3CA*-activating mutations are associated with early recurrence and poor prognosis in glioblastoma. Our dataset included patients who received identical adjuvant treatment in a single center, and our early recurrence finding was validated in a large, multicenter independent dataset [19]. Additionally, we identified novel clinical associations with *PIK3CA*-activating mutations, including younger age at diagnosis and propensity to present with widespread disease. We demonstrated that *PIK3CA*-activating mutations were associated with clinically-apparent increased invasiveness and/or CNS dissemination in patients with newly diagnosed glioblastoma.

Increased PI3K signaling is well known to promote the survival, growth, and proliferation of cancer cells [9, 11], as well as their motility, invasion, and metastasis [9, 25, 26]. In vitro studies have similarly demonstrated that inappropriately activated PI3K signaling was associated with cell motility and invasion in glioma [27–30]. Recently, Lee et al. reported that multifocal glioblastomas were enriched for *PIK3CA* mutations [31], which is consistent with our findings. Therefore, we speculate that activated *PIK3CA* drives increased glioma cell migration, resulting in a disseminated malignant phenotype that may escape standard-of-care adjuvant involved-field radiation therapy. In the TCGA validation dataset, *PIK3CA* mutations were associated with shorter PFS, but the association with OS was not statistically significant. The greater likely patient variation in the larger TCGA dataset and wide variety of salvage therapies for the recurrent disease could partially account for the lack of significance seen in OS analysis.

There were some limitations to this study. The sample size of *PIK3CA* mutations was small; however, the mutation frequency in our large cohort was consistent with the findings of previous studies [15], and the demographic and molecular characteristics as well as treatment and outcomes in our entire cohort were largely comparable to those in other large published datasets on glioblastoma [17, 32], indicating the generalizability of our cohort. The progression of glioblastoma may be difficult to discern from treatment-related necrosis radiographically; however, all the progression events in this study were well-evidenced by standard response assessment criteria [33, 34] and confirmed by well-documented clinical and radiographic follow-up.

Another concern is the lack of assessment on other potentially important genes in the SNaPshot versions used in this study, including *IDH2*, *PIK3R1* and *TERT* promoter [35]. *IDH2* is present in only 2% of all diffuse gliomas and less than 1% in adult primary glioblastoma – the study population in this study [32], therefore including *IDH2* would not likely impact the results. *PIK3R1* mutations have been shown to induce a gain of PI3K enzymatic function and enhance PI3K signaling, which implies that *PIK3R1* mutant glioblastomas may well have the similar impact as the *PIK3CA* mutant counterpart. However, the aforementioned study by Lee et al. demonstrated that *PIK3CA* mutations were enriched in multifocal glioblastomas, while *PIK3R1* mutations were not [31]. *PIK3CA* mutations were associated with shorter PFS on the TCGA dataset, but *PIK3R1* mutations were not. These observations may suggest a differential impact on clinical pictures of glioblastomas between the two PI3K pathway mutations. The prognostic implication of *PIK3R1* mutation remains to be elucidated and definitely warrants further studies. A recent study by Izquierdo et al. reported on radiological characteristics of *IDH* wildtype lower grade astrocytoma [36]. *TERT* promoter mutations in this cohort were associated with glimatosis-like pattern at presentation, and apparently tended for poorer prognosis. Our recent study investigated on a more recent cohort from our institution demonstrated that *TERT* promoter wildtype glioblastoma, *IDH* wildtype harbored frequent PI3K pathway mutations as compared to the *TERT* promoter mutant counterpart [35]. This study lacked radiological assessment or survival follow-up. Therefore, the interactions between *PIK3R1* mutation, *TERT* promoter mutation and *PIK3CA* mutation in adult glioblastoma clinical characteristics remain to be determined.

This study confirms our initial observation that *PIK3CA* mutant glioblastomas were associated with more widespread disease at presentation and shorter PFS [37] as well as a recent report by Lee et al. [31], who reported *PIK3CA* mutations were associated with multifocal/multicentric (versus solitary) enhancing tumors and decreased survival. In addition, we extend Lee et al.’s cohort to include distant leptomeningeal spread and non-enhancing gliomatosis. Based on these data, and the finding by Lee et al. that *PIK3CA* mutation is early and truncal in glioblastoma [31], knowledge of *PIK3CA* mutation status could aid in the decision of whether to utilize locally-directed therapies such as surgical resection and focal radiation therapy. Additionally, clinical trial designs may have to account for *PIK3CA* mutation status, as widespread or multifocal disease often precludes patients from participating in clinical trials. Inhibitors of PI3K and mammalian target of rapamycin tested in trials are theoretically the most effective for tumors addicted to PI3K signaling, such as *PIK3CA* mutant tumors, however patients with these tumors may be preferentially excluded from clinical trials because of their widespread disease. Therefore, our findings may have significant implications for interpreting the results of clinical trials of PI3K inhibitors [38] and for designing PI3K-specific clinical trials (NCT01339052).

## Conclusions

This study indicates that *PIK3CA*-activating mutations identify a subset of glioblastomas associated with younger patient age, early progression, and propensity to present with widespread disease. Patients with *PIK3CA* mutant glioblastoma may require additional consideration in treatment planning and clinical trials.

## Additional files

Additional file 1: **Figure S1.** Kaplan-Meier curves of OS and PFS stratified by established molecular prognostic factors (*IDH1* mutation and *MGMT* promoter methylation). PFS (A and C) and OS (B and D) are plotted stratified by *IDH1* mutation (A and B) and *MGMT* promoter methylation (C and D). The curves shown in solid lines represent *IDH1* mutant or *MGMT* promoter methylated and those in dashed lines represent *IDH1* wildtype or *MGMT* promoter unmethylated. (TIF 158 kb)

Additional file 2: **Figure S2.** Kaplan-Meier curves of OS stratified by *PIK3CA* mutation. *PIK3CA* mutant tumors (solid) and wildtype tumors (dashed) depicted. (TIF 493 kb)

Additional file 3: **Figure S3.** Kaplan-Meier curves of PFS in *IDH1* wildtype glioblastomas stratified by *PIK3CA* mutation. *PIK3CA* mutant tumors (solid) and wildtype tumors (dashed) depicted. (TIF 487 kb)

Additional file 4: **Figure S4.** Kaplan-Meier curves of OS in *IDH1* wildtype glioblastomas stratified by *PIK3CA* mutation. *PIK3CA* mutant tumors (solid) and wildtype tumors (dashed) depicted. (TIF 493 kb)

Additional file 5: **Figure S5.** Kaplan-Meier curves of PFS stratified by *PIK3CA* mutation in the TCGA cohort. *PIK3CA* mutant (solid) and *PIK3CA* wildtype (dashed) glioblastoma patients depicted. (TIF 494 kb)

Additional file 6: **Table S1.** Association of *PIK3CA* mutation with progression-free and overall survival in *IDH1* wildtype glioblastoma. (DOC 55 kb)

## Abbreviations

EGFR: Epithelial growth factor receptor
FISH: Fluorescence in situ hybridization
IDH1: Isocitrate dehydrogenase
KPS: Karnofsky performance status
MET: Mesenchymal-epithelial transition
MGMT: O^6^-methylguanine-DNA methyltransferase
OS: Overall survival
PDGFR: Platelet-derived growth factor receptor alpha
PFS: Progression-free survival
PI3K: Phosphatidylinositol 3-kinase
PIK3CA: Phosphatidylinositol-4,5-bisphosphate 3-kinase catalytic subunit alpha
PIK3R1: Phosphatidylinositol-4,5-bisphosphate 3-kinase regulatory subunit 1
TCGA: The Cancer Genome Atlas
