# Supplementary figures and images for: PIK3CA activating mutations are associated with more disseminated disease at presentation and earlier recurrence in glioblastoma
Source: Acta Neuropathol Commun. 2019 Apr 29;7:66. doi: 10.1186/s40478-019-0720-8 (PMC6487518; doi:10.1186/s40478-019-0720-8)

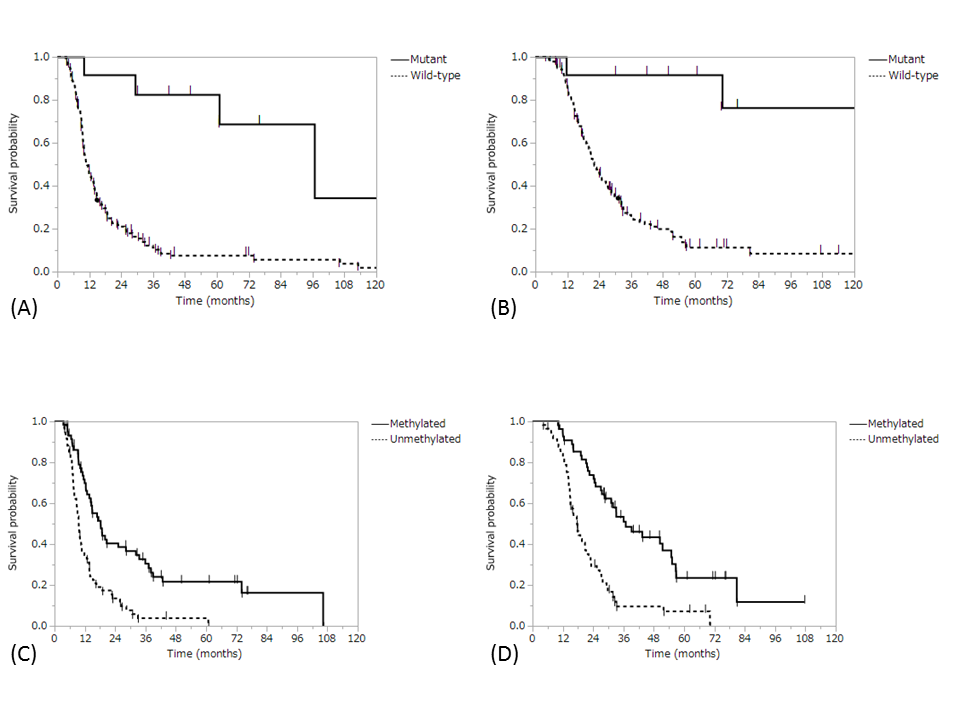

Supplement: Supplementary file 1 — Figure S1. Kaplan-Meier curves of OS and PFS stratified by established molecular prognostic factors (IDH1 mutation and MGMT promoter methylation). PFS (A and C) and OS (B and D) are plotted stratified by IDH1 mutation (A and B) and MGMT promoter methylation (C and D). The curves shown in solid lines represent IDH1 mutant or MGMT promoter methylated and those in dashed lines represent IDH1 wildtype or MGMT promoter unmethylated. (TIF 158 kb) [file 40478_2019_720_MOESM1_ESM.tif]

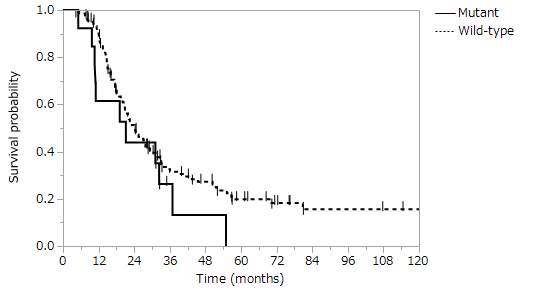

Supplement: Supplementary file 2 — Figure S2. Kaplan-Meier curves of OS stratified by PIK3CA mutation. PIK3CA mutant tumors (solid) and wildtype tumors (dashed) depicted. (TIF 493 kb) [file 40478_2019_720_MOESM2_ESM.tif]

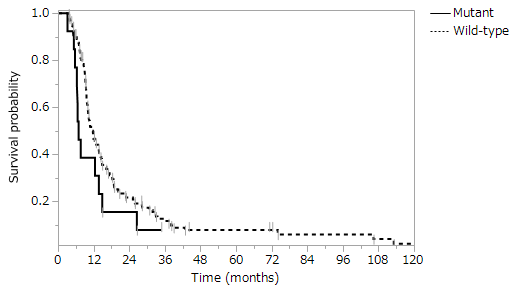

Supplement: Supplementary file 3 — Figure S3. Kaplan-Meier curves of PFS in IDH1 wildtype glioblastomas stratified by PIK3CA mutation. PIK3CA mutant tumors (solid) and wildtype tumors (dashed) depicted. (TIF 487 kb) [file 40478_2019_720_MOESM3_ESM.tif]

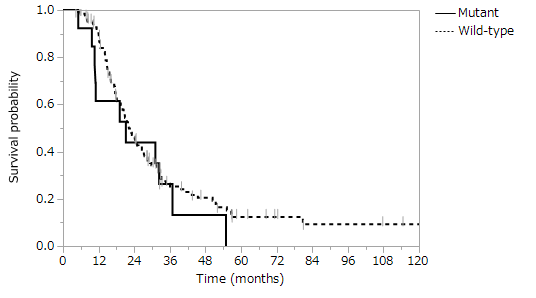

Supplement: Supplementary file 4 — Figure S4. Kaplan-Meier curves of OS in IDH1 wildtype glioblastomas stratified by PIK3CA mutation. PIK3CA mutant tumors (solid) and wildtype tumors (dashed) depicted. (TIF 493 kb) [file 40478_2019_720_MOESM4_ESM.tif]

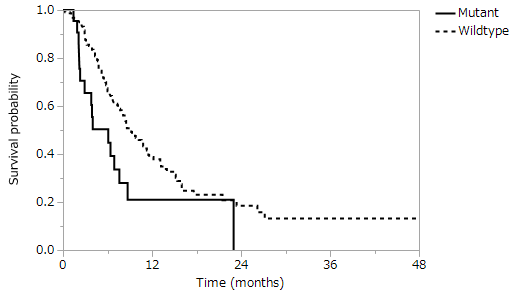

Supplement: Supplementary file 5 — Figure S5. Kaplan-Meier curves of PFS stratified by PIK3CA mutation in the TCGA cohort. PIK3CA mutant (solid) and PIK3CA wildtype (dashed) glioblastoma patients depicted. (TIF 494 kb) [file 40478_2019_720_MOESM5_ESM.tif]
