# Supplementary material for: PIK3CA activating mutations are associated with more disseminated disease at presentation and earlier recurrence in glioblastoma
Source: Acta Neuropathol Commun. 2019 Apr 29;7:66. doi: 10.1186/s40478-019-0720-8 (PMC6487518; doi:10.1186/s40478-019-0720-8)
Supplement: Supplementary file 6 — Table S1. Association of PIK3CA mutation with progression-free and overall survival in IDH1 wildtype glioblastoma. (DOC 55 kb) [file 40478_2019_720_MOESM6_ESM.doc]

**Additional file 6: Table S1.** Association of *PIK3CA* mutation with progression-free and overall survival in *IDH1* wildtype glioblastoma.

[PFS]

|  | Univariate analysis | Multivariate analysis | |
| --- | --- | --- | --- |
|  | *P* value | *P* value | HR |
| High age | 0.13 | 0.006^*^ | 1.03 (1.01-1.05)^#^ |
| High KPS score | 0.28 | 0.30 | 0.99 (0.97-1.01)^#^ |
| Gross total resection | 0.03^*^ | 0.15 | 0.74 (0.48-1.11) |
| *PIK3CA* mutation | 0.11 | 0.01^*^ | 2.85 (1.28-5.83) |
| *MGMT* promoter methylation | <0.0001^*^ | <0.0001^*^ | 0.35 (0.22-0.54) |
| * Statistical significance: *P*≤0.05 |  |  |  |
| ^#^ HR per unit | | | |
|  | | | |

[OS]

|  | Univariate analysis | Multivariate analysis | |
| --- | --- | --- | --- |
|  | *P* value | *P* value | HR |
| High age | 0.052 | 0.005^*^ | 1.03 (1.01-1.05)^#^ |
| High KPS score | 0.10 | 0.34 | 0.99 (0.97-1.01)^#^ |
| Gross total resection | 0.12 | 0.50 | 0.86 (0.54-1.33) |
| *PIK3CA* mutation | 0.42 | 0.057 | 2.25 (0.97-4.73) |
| *MGMT* promoter methylation | <0.0001^*^ | <0.0001^*^ | 0.29 (0.18-0.47) |
| * Statistical significance: *P*≤0.05 | | | |
| ^#^ HR per unit | | | |

Abbreviations: PFS, progression-free survival; OS, overall survival; KPS, Karnofsky performance status; *IDH1*, isocitrate dehydrogenase 1; *MGMT*, O^6^-methylguanine-DNA methyltransferase; HR, hazard ratio
